# Treatment of fevers prior to introducing rapid diagnostic tests for malaria in registered drug shops in Uganda

**DOI:** 10.1186/1475-2875-12-131

**Published:** 2013-04-16

**Authors:** Anthony K Mbonye, Sham Lal, Bonnie Cundill, Kristian Schultz Hansen, Siân Clarke, Pascal Magnussen

**Affiliations:** 1 School of Public Health, Makerere University and Commissioner Health Services, Ministry of Health, Box 7272, Kampala, Uganda; 2 Department of Disease Control, London School of Hygiene and Tropical Medicine, Keppel Street, London, WC1E 7HT, UK; 3 Department of Infectious Disease Epidemiology, London School of Hygiene and Tropical Medicine, Keppel Street, London, WC1E 7HT, UK; 4 Department of Global Health and Development, London School of Hygiene and Tropical Medicine, 15-17 Tavistock Place, London, WC1H 9SH, UK; 5 Centre for Medical Parasitology, University of Copenhagen, Copenhagen, Denmark

## Abstract

**Background:**

Since drug shops play an important role in treatment of fever, introducing rapid diagnostic tests (RDTs) for malaria at drug shops may have the potential of targeting anti-malarial drugs to those with malaria parasites and improve rational drug use. As part of a cluster randomized trial to examine impact on appropriate treatment of malaria in drug shops in Uganda and adherence to current malaria treatment policy guidelines, a survey was conducted to estimate baseline prevalence of, and factors associated with, appropriate treatment of malaria to enable effective design and implementation of the cluster randomized trial.

**Methods:**

A survey was conducted within 20 geographical clusters of drug shops from May to September 2010 in Mukono district, central Uganda. A cluster was defined as a parish representing a cluster of drug shops. Data was collected using two structured questionnaires: a provider questionnaire to capture data on drug shops (n=65) including provider characteristics, knowledge on treatment of malaria, previous training received, type of drugs stocked, reported drug sales, and record keeping practices; and a patient questionnaire to capture data from febrile patients (n=540) exiting drug shops on presenting symptoms, the consultation process, treatment received, and malaria diagnoses. Malaria diagnosis made by drug shop vendors were confirmed by the study team through microscopy examination of a blood slide to ascertain whether appropriate treatment was received.

**Results:**

Among febrile patients seen at drug shops, 35% had a positive RDT result and 27% had a positive blood slide. Many patients (55%) had previously sought care from another drug shop prior to this consultation. Three quarters (73%) of all febrile patients seen at drug shops received an anti-malarial, of whom 39% received an ACT and 33% received quinine. The rest received another non-artemisinin monotherapy. Only one third (32%) of patients with a positive blood slide had received treatment with Coartem^®^ while 34% of those with a negative blood slide had not received an anti-malarial. Overall appropriate treatment was 34 (95% CI: 28 – 40) with substantial between-cluster variation, ranging from 1% to 55%.

**Conclusion:**

In this setting, the proportion of malaria patients receiving appropriate ACT treatment at drug shops was low. This was due to the practice of presumptive treatment, inadequate training on malaria management and lack of knowledge that Coartem^®^ was the recommended first-line treatment for malaria. There is urgent need for interventions to improve treatment of malaria at these outlets.

## Background

In Uganda, malaria is highly endemic and is the leading cause of morbidity and mortality [1,2]. The main component of the current malaria control strategy in Uganda is early diagnosis and effective case management. In Uganda and elsewhere, effective treatment of fever has been hampered by inappropriate treatment practices, particularly in rural areas where there is poor access to formal health facilities and self-treatment is the commonest form of care-seeking [3-7]. Inappropriate treatment practices include, on the one hand, the continuing over-diagnosis of malaria and over-treatment with anti-malarial drugs among patients presenting with a fever [8-10], constituting an unnecessary waste of resources. On the other hand, misdiagnosis can lead to non-malaria febrile illnesses remaining untreated, with associated risks for the patient.

The treatment of febrile illnesses in malaria-endemic countries has received increasing attention in the past decade, with most countries in sub-Saharan Africa changing the first-line anti-malarial treatment to artemisinin-based combination therapy (ACT). The higher cost of ACT, together with increasing recognition of the importance of non-malarial fevers have contributed to reconsideration of existing strategies on presumptive treatment of fevers. Accordingly, WHO has recommended targeted treatment of malaria at all levels of health care, including the private sector [11]. Rapid diagnostic tests (RDTs) for malaria provide a means of confirming malaria diagnosis, particularly in remote locations lacking electricity for microscopes and qualified health staff. RDTs are affordable, quick, accurate and relatively easy to perform with minimal training [12-15].

Although there has been a rapid increase in the roll-out of RDTs in public health facilities, the introduction of RDTs in the private sector has been much slower. The private sector, in particular drug shops play an important role in provision of healthcare with up to 80% of malaria cases treated outside the formal health sector [9]. In many cases, drug shops are frequently visited as the first (often only) source of treatment because they are numerous, easily accessible, and more oriented to satisfying consumer needs [9,10]. A recent survey in Uganda suggests that an estimated 50% of all anti-malarial drugs are distributed through drug shops [16]. Hence, the introduction of RDTs in drug shops has the potential to make a significant contribution to targeting anti-malarial drugs to those with malaria parasites and improve rational drug use. Although RDTs were introduced into Uganda at lower health facilities in 2010 [17], they are not widely implemented due to inadequate funds to avail them.

Although there is limited evidence of the best way to scale up RDTs across sectors, there is a suggestion that public providers do not always adhere to the test results continuing to prescribe anti-malarial drugs to patients with a negative RDT result [18-20]. However, supportive interventions for provider behaviour change, particularly supportive supervision, have been reported to be effective in restricting anti-malarial drugs to parasite positive patients [21-24]. There is less evidence from the private sector and the best strategies to promote RDTs in this sector remain unclear.

In light of the strong need for more research on improving case management of malaria in the private sector, a cluster randomized trial to examine the feasibility and cost-effectiveness of an intervention to introduce RDTs into registered drug shops in Uganda, with a focus on the correct use and adherence to ACT, was initiated.

The data presented here are from baseline surveys undertaken as part of the formative research with the objectives of determining the prevalence of appropriate treatment for malaria and misuse of anti-malarial drugs at baseline; to characterize patients, drug shops and providers to inform the design of the intervention; and to explore associations between these characteristics and treatments sold, under a situation where presumptive diagnosis of malaria was the main practice. The results from the qualitative component of the formative research have previously been reported elsewhere [25,26].

## Methods

### Study area and population

Baseline surveys were conducted within clusters of drug shops eligible for inclusion in an upcoming randomized trial of RDT-based diagnosis in Mukono, Central Uganda, a district hyperendemic for malaria, with transmission peaks in May-June and November-December. The total population of the district is 850,900 with an annual growth rate of 2.3% and consists of predominantly subsistence farmers of the Baganda ethnic group. The majority of the population, 88%, lives in the rural areas.

A list of all 63 parishes in Mukono district was obtained from Uganda Bureau of Statistics, and reviewed against the inclusion criteria. Parishes were deemed eligible for inclusion in the trial if they: i) contained a health centre II, the lowest public health facility where early treatment is sought; ii) contained more than 200 households to ensure a sufficient number of patients visiting the drug shops; and iii) contained at least one registered drug shop registered with the District Drug Inspector (DDI). All registered drug shops in the eligible parishes were mapped using a global positioning system (GPS), Garmin Corporation, and grouped into clusters. A cluster was defined as a natural grouping of drug shops which was often synonymous with a single parish, a geographical demarcated area covering a population of approximately 5,000 people, but could include drug shops in a neighbouring parish where the distance between drug shops in different parishes was <1 km. A total of 20 clusters fulfilled the eligibility criteria and all were included in the study.

Prior to baseline data collection, community sensitization activities involving village health teams were conducted throughout the study area to raise awareness on the upcoming cluster randomized trial in registered drug shops and associated research activities.

### Data collection

Baseline surveys were conducted via structured questionnaires with i) drug shop owners and ii) patients exiting drug shops, in all 20 clusters between May and September 2010. Within each cluster, drug shops owners from all registered drug shops were invited to be included in the survey, and written consent was obtained. Interviews with providers captured details of the registration, provider characteristics, knowledge on treatment of malaria, previous training received, anti-malarial drugs stocked, drug sales, patient records kept and opening hours.

A patient exit survey was conducted, in which all patients exiting the drug shops, who had presented with fever or a history of fever, were invited to participate in a structured questionnaire interview. Written informed consent from patients was sought prior to interview. Data was captured on the presenting illness and symptoms, treatment received, including anti-malarial drugs purchased and how malaria was diagnosed at the drug shops. Five social scientists experienced in malaria research conducted the interviews. They underwent refresher training for 5 days on qualitative research techniques, and study procedures; and participated in the pretesting and revision of the questionnaire tools before actual field work. Questionnaires were administered in the local language (Luganda). Site supervisors monitored and supervised all aspects of data collection.

Patients were also asked if they would consent to provide a finger-prick blood sample at the end of the interview for confirmation of malaria parasites. Blood samples were taken by a trained laboratory technician who prepared a thick blood film for microscopy and performed an RDT (First Response^®^). The RDT test was read in the field and patients told the outcome. Blood smears were stained with Giemsa and parasites were counted against 200 leukocytes and expressed as number of parasites per μl of blood assuming a standard leukocyte count of 8,000/μl of blood. A blood smear was regarded negative after examining a minimum of 100 high power fields with no parasites seen. Sample size calculations indicated that a harmonic mean of 25 patients sampled per cluster would allow estimation of the proportion of patients receiving appropriate treatment for malaria with a precision of ±10%, assuming an intra-cluster correlation of 0.2 and a prevalence of 50% for the outcome of interest [27].

### Statistical methods

Data was entered and verified using Microsoft Access 2007 (Microsoft Inc., Redmond, Washington) and analysed using STATA version 11.0 (STATA Corporation, College Station, Texas). The proportion of correctly treated malaria cases was based on the microscopy reading as a “gold standard”. Appropriate treatment was defined as a composite proportion including febrile patients (denominator) that are a) slide-positive and are given a first-line anti-malarial drug; and b) slide negative patients who do not get an anti-malarial. Socio-economic status was measured based on reported ownership of household assets included in national census, and a wealth index generated through principal components analysis [28], which was subsequently divided into quintiles.

Treatment outcomes and 95% CIs were estimated allowing for the survey design in STATA by identifying sampling weights and clustering of patients within drug shop clusters. Differences between percentages were compared using a t-test. Random effects logistic regression was used to identify factors associated with appropriate treatment at drug shops. Factors (age, sex, education level, marital status, wealth index, treatment seeking elsewhere, length of illness and staff characteristics) with a strong association (odds ratio <0.5 or >1.5) with appropriate treatment or a *P*-value less than 0.10 identified in univariate analyses were entered into a multivariable model using a forward stepwise selection method. Factors were retained in this multivariable model if they remained statistically associated at the 10% level. Models were compared using a log-likelihood ratio test.

### Ethics

Ethical approval for the research was granted from review boards at the Uganda National Council of Science and Technology and the London School of Hygiene and Tropical Medicine. Written informed consent was sought prior to the interviews.

## Results

### Interviews with treatment providers at drug shops

#### Characteristics of registered drug shops

There were 65 registered drug shops within the 20 clusters eligible for inclusion in the study with a range of one to seven shops per cluster. All drug shop owners consented to participate in the study. Forty-six (71%) drug shops were registered with the National Drug Authority (NDA), the remainder with the District Drug Inspectorate (DDI). Three drug shops (4.6%) were located in rural areas of the district, the rest were predominately based in rural trading centres with 13 rural (21%), and urban townships (79%). All drug shops were open at least five days a week, with the majority (68%) open every day. Typically drug shop vendors reported seeing between 2–50 febrile patients (10 patients on average) each day, with the largest number of patients visiting in the evening between 5-7 pm.

#### Staff characteristics and training

The majority (80%) of drug shop staff were female and educated to secondary level (97%). Although many drug shop operators (68%) were qualified health workers; less than half (46%) had received any training on management of malaria in the last 5 years, such as ACT or Integrated Management of Childhood Illness (IMCI) guidelines; none had training in microscopy and only 5% had been trained on RDTs (Table 1). When their knowledge on the current malaria treatment policy and RDTs was assessed, 42 (65%) drug shop staff claimed that they knew the recommended first-line anti-malarial treatment, but only 43% (28/65) specified an ACT, such as Coartem^®^; whilst 11 could not specify any drug. Of the 42 claiming knowledge of the treatment policy, 15 (36%) reported they had learned about the first-line treatment from the national guidelines; 11 (26%) from another health worker; five (12%) from training workshops; while the remainder stated that they had learned about the first-line treatment from the DDI, the radio or general conversation. Regarding RDTs, only 19 of the 65 drug shop operators interviewed (29%) knew what an RDT was for; of whom 10 had learned about RDTs from another health worker; 6 from the national guidelines; while 3 had heard about RDTs from the research team as part of community sensitization/consent process. Only four drug shops (6%) had a copy of the first-line malaria treatment policy; two drug shops (3%) had additional posters and leaflets available on ACT, and only one had any information/material available on RDTs for customers.

**Table 1:** Characteristics of staff in registered drug shops

**Staff characteristics (N=65)**	**N (%)**
**Sex**	
Male	13 (20%)
Female	52 (80%)
**Education**	
Primary	0 (0%)
Secondary	63 (97%)
Tertiary (Technical/University)	2 (3%)
**Professional training**	
**High Level of Training**	30 (46%)
Enrolled Nurse	20
Midwife	5
Clinical Officer	3
Comprehensive nurse	1
Psychiatric nurse	1
**Low Level of Training**	35 (54%)
Nursing Aide	21
Auxiliary Nurse	14
**Knowledge of first line malaria treatment**	
No	37 (57%)
Yes	28 (43%)
**Training in malaria case management **^**‡**^	
Artemisinin Combination Therapy (ACT)	18 (28%)
IMCI Guidelines^§^	12 (18%)
Rapid diagnostic tests (RDTs)	3 (5%)
Microscopy	0 (0%)
None of the above training	39 (60%)
**Knowledge of RDT**	
Does not know	46 (71%)
Knows	19 (29%)

Notes:

^‡ ^more than one type of training possible.

^§ ^IMCI stands for Integrated Management of Childhood Illness [29].

#### Diagnosis and treatment of malaria at drug shops

**Figure 1:**
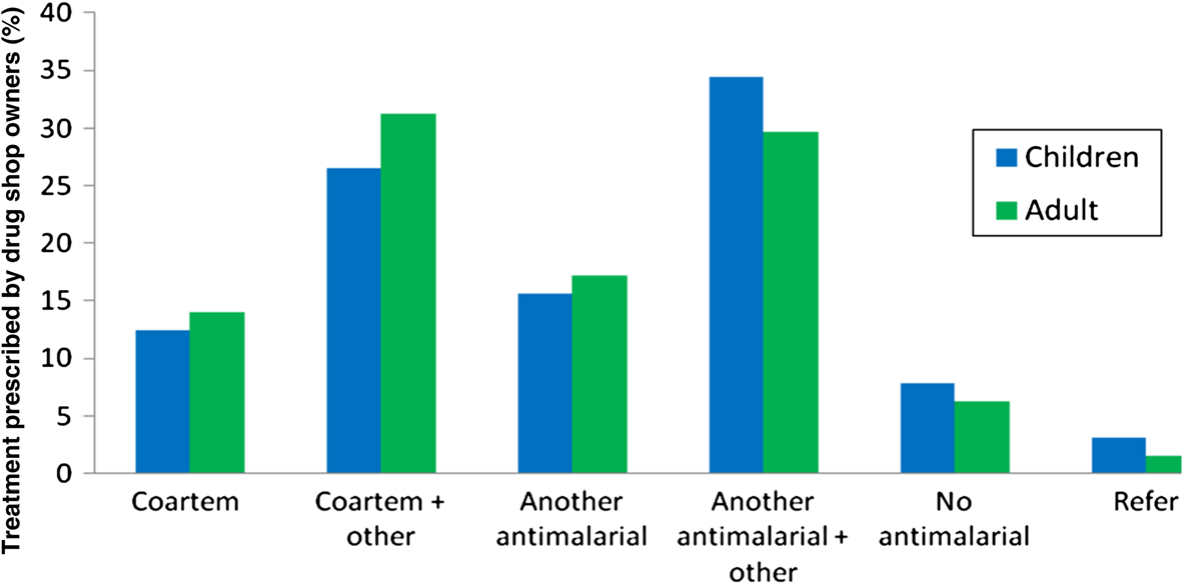
Treatment prescribed to adults and children who present with fever by registered drug shop staff.

Thermometers were available in the majority of shops (58/65, 89%), while only four (6%) had a functioning microscope and offered malaria testing. Three quarters (75%) of shops stocked Coartem^®^, the recommended first-line treatment for malaria in Uganda. Other anti-malarial drugs stocked included quinine (89%), Fansidar^®^ (89%), Camoquine^®^ (54%) and chloroquine (38%). None of the drug shops stocked artemisinin monotherapies. When drug shop vendors were asked what treatment they prescribe to a febrile child, the most frequently reported anti-malarial prescribed was quinine (44%), often in conjunction with another medication, such as Fansidar, paracetamol tablets and syrup. Only 40% said they would prescribe Coartem^®^, again often given in combination with paracetamol or an antibiotic; 8% did not prescribe any anti-malarial, instead they prescribed paracetamol and/or antibiotics. A similar prescribing pattern was reported for adults (Figure 1). Over four-fifths (53/64, 83%) of drug shops reported seeing patients with severe illness; with an average of two patients with severe illness seen per week. All but one drug shop vendor reported referring patients with severe illness; 32 (62%) referred to a health centre, 17 (33%) to a hospital. Although two-fifths (38%) of drug shop vendors stated that they did not encounter any constraints in referring patients, the majority did report constraints to referral. Lack of money was identified by drug shop vendors as the biggest hindrance to referral (Table 2).

**Table 2:** Constraints reported by drug shop staff when referring patients

**Perceived constraint (N=65)**	**N (%)**
No perceived constraints	25 (38%)
One or more constraints reported*	40 (62%)
Patients do not have the money	30
Patients do not comply (reasons unknown)	22
No drugs at the referral facility	15
Referral facilities are too far	11
Waiting time at health centre	2
No stamps on referral letters	1
Patient doubt your advice	1
Severe symptoms	1

* More than one response possible.

### Exit interviews with patients at drug shops

Exit interviews were conducted with a total of 540 clients seeking treatment for fever; none of those approached refused to participate in the exit interview. The number of patients interviewed per drug shop ranged between five to 68 patients; and all patients’ (or their caregivers) present at the drug shop consented to provide a blood slide (n=540) (data not shown). The characteristics of the patients interviewed are presented in Table 3. Patients’ age ranged from 1 month to 76 years; 35% were under the age of 5 years. In the majority of cases, the patients who were sick came to the drug shop in person, and only 5% of patients were seeking treatment for someone else. When asked about symptoms, two-fifths (41%) of patients interviewed also reported a cough alongside the fever; other co-presenting symptoms included common cold (34%), headache (19%), abdominal pain (14%) and diarrhoea (10%) (Table 3).

**Table 3:** Characteristics of febrile patients exiting registered drug shops

**Patient characteristics**	**Frequency**
**Age (years), N=540**	
<5	190 (35%)
5-14	120 (22%)
≥15	230 (43%)
**Sex, N=540**	
Male	248 (46%)
Female	292 (54%)
**Pregnant**^**†**^**, N=127**	
Yes	9 (7%)
No	118 (93%)
**Education (or caregiver education)**^**‡**^**, N=353**	
None	45 (13%)
Primary	196 (55%)
Secondary	99 (28%)
Tertiary (Technical/University)	13 (4%)
**Marital status**^**§ **^**n=533**	
Single	375 (71%)
Married/Cohabiting	129 (24%)
Widowed/Separated	29 (5%)
Wealth index* n=539	
Poorest	210 (39%)
Less poor	164 (30%)
Least poor	165 (31%)
**Presenting symptoms**^**#**^	
Cough	224 (41%)
Common cold	184 (34%)
Headache	100 (19%)
Abdominal pain	74 (14%)
Diarrhoea	56 (10%)

Notes:

^† ^Only asked among females aged 15 years and over (n=127).

^‡ ^Not all customers interpreted the question as the education of the patient or caregiver if the patient is a child. Hence results on available for 353 patients.

^§ ^Marital status is not known for 7 patients.

*Wealth index was not known for 1 patient. Variables included in the calculation of the wealth index were household occupation, housing type, ownership of radio, bicycle, boda boda (motorbike), car, shop.

^#^ Patients may have presented with more than one symptom.

**Figure 2:**
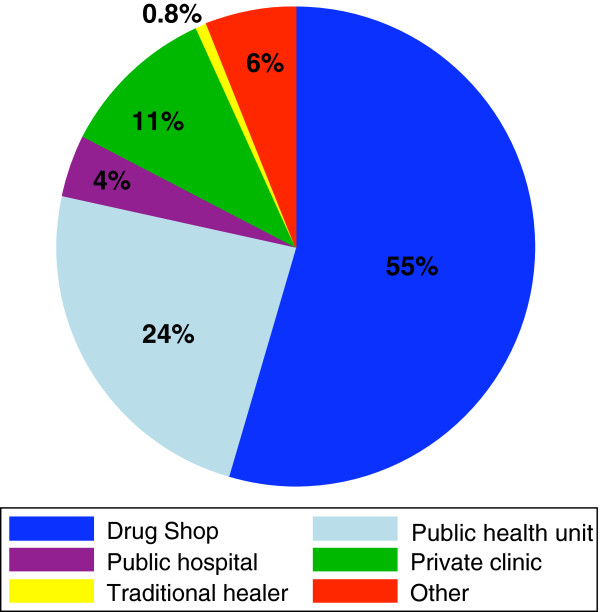
Sources of previous treatment before visiting the drug shop.

In over half of the exit interviews, patients reported that they chose this particular drug shop for treatment because they had been there before (61%) and because the provider was friendly (54%). Other reasons given included proximity of the drug shop to their home (38%), because the provider was qualified (26%), ability to get treatment on credit (25%) and availability of drugs (23%). The median number of days from the start of illness to the time of visiting a drug shop was 3 days; and just under half (48%) had sought treatment elsewhere before visiting the drug shop where they were interviewed. The most popular source of previous treatment was another drug shop (Figure 2).

### Prevalence of malaria and appropriate treatment

Of the 494 patients (91%) who were told the name of their illness by the drug shop vendor, three quarters (387/494) reported having been told they had malaria. However, results from the examination by the research team of blood samples provided after the exit interviews showed that only 190 (35%) of the 538 (2 results missing) had a positive RDT while 145/540 (27%), had a positive blood slide. About three quarters (73%) of patients received an anti-malarial, of which 39% received an ACT, 33% received quinine, and the rest another non-artemisinin monotherapy. In shops which did not have any ACT in stock, quinine was the most commonly prescribed anti-malarial (Table 4). Of even greater concern was the finding that in shops that did stock ACT, almost a third of the patients that were later found to be blood-slide positive (40/128, 31%) had not received an ACT. Among patients interviewed and found to have a positive blood slide by microscopy, 32% (95% CI: 20-45%) had received treatment with Coartem^®^ from the drug shop, but 17% (95% CI: 10–23) received no anti-malarial at all. Among those with a negative blood slide, a third (95% CI: 27-42%) had not received an anti-malarial but 66% (95% CI: 58-73%) had needlessly received an anti-malarial. Overall, appropriate treatment by drug shop vendors in the absence of RDT testing was estimated at 34% (95% CI 28 – 40%) with substantial between-cluster variation, ranging from 1% to 55%. Treatment with antibiotics was similar among those with and without confirmed malaria and in drug shops with and without ACT in stock (Table 4).

**Table 4:** Treatment by diagnosis results and drug availability

	**Availability of ACT**							**Microscopy (blood slide result)**				
**Treatment received**	**No of patients**	**% (95% CI)**	**ACT in stock**	**% (95% CI)**	**ACT not in stock**	**% (95% CI)**	**p-value**	**Positive**	**% (95% CI)**	**Negative%**	**% (95% CI)**	**p-value**
**Coartem (ACT)**	154	27 (19–35)	154	100	0		n/a	53	32 (20 – 45)	101	26 (17 – 34)	0.29
**Quinine**	131	26 (18 – 33)	73	19 (16 – 22)	58	52 (38 – 66)	<0.01	46	36 (26 – 47)	85	22 (15 – 29)	<0.01
**Fansidar**	71	11 (5 – 17)	51	11 (5 – 17)	20	12 (0.1 – 24)	0.84	16	11 (5–19)	55	9 (4 – 14)	0.17
**Camoquine**	27	5 (2 – 8)	18	5 (1 – 8)	9	6 (3 – 8)	0.61	6	4 **(2–6)**	21	5 (0.1 – 9)	0.73
**Chloroquine**	14	2 (0.1 – 4)	12	2 (0.3 – 4)	2	1 (-1 – 3)	0.44	4	2 (-0.2 – 4)	10	2 (0.4 – 3.6)	0.95
**No anti-malarial**	148	30 (23 – 37)	305	74 (70 – 78)	87	68 (60 – 76)	0.20	123	83 (77 – 90)	269	66 (58 – 73)	<0.01
**Antibiotic**	151	31 (22 – 40)	105	29 (18 – 39)	46	40 (35 – 46)	0.03	43	33 (14 – 53)	108	30 (24 – 36)	0.68
**Paracetamol**	385	69 (61 – 76)	293	68 (60 – 78)	92	70 (61 – 77)	0.93	115	78 (64 – 92)	270	65 (59 – 72)	0.03

Appropriate treatment.

34 (28 – 40).

In univariable analysis, patients who had a higher wealth index (*P*=0.03) and increasing duration of illness (*P*=0.03) were more likely to be treated appropriately. There was no evidence that factors such as training on malaria and staff training were associated with appropriate treatment. In multivariable analysis, patient’s household wealth, length of a patient’s illness remained independently associated with receiving appropriate treatment (Table 5).

**Table 5:** Factors associated with access to appropriate treatment at drug shops

		**Univariable analysis**		**Multivariable analysis**	
**Patient characteristics**	**n**	**OR (95% CI)**	***p*-value**	**OR (95% CI)**	***p*-value**
**Sex**					
Male	248	1	0.84		
Female	292	0.96 (0.67, 1.39)			
**Age (years)**					
<5	190	1	0.44		
5-14	120	1.37 (0.84, 2.25)			
≥ 15	230	1.17 (0.77, 1.79)			
**Education (or caregiver education)**					
None	45	1	0.34		
Primary	196	1.65 (0.78, 3.51)			
Secondary	99	1.90 (0.84, 4.27)			
Tertiary (Technical/University)	13	0.84 (0.19, 2.73)			
**Marital status**					
Single	375	1	0.32		
Married/Cohabiting	129	0.81 (0.52, 1.26)			
Widowed/Separated	29	1.52 (0.69, 3.34)			
**Wealth index**					
Poorest	210	1	0.03	1	0.03
Less poor	164	0.59 (0.37, 0.93)		0.62 (0.38 – 0.99	
Least poor	165	1.08 (0.70, 1.67)		1.13 (0.72 – 1.75)	
**Previously sought treatment elsewhere**					
No	283	1	0.27		
Yes	257	1.23 (0.85, 1.78)			
**Length of illness (hours/days)**					
≤24 hours	56	1	0.03	1	0.04
24-48 hours	133	1.78 (0.85, 3.72)		1.73 (0.82 – 3.63)	
3 – 7 days	299	1.61 (0.81, 3.21)		1.51 (0.76 – 3.02)	
> 7 days	52	3.49 (1.49, 8.16)		3.25 (1.38 -7.66)	
**Health worker characteristics**					
**Staff sex**					
Male	13	1			
Female	52	0.83 (0.50, 1.37)	0.46		
**Staff Training**					
High level of training	30	1			
Low level of training	35	0.85 (0.57, 1.26)	0.41		
**Knowledge first line treatment is ACT**					
No	37	1			
Yes	28	1.08 (0.73, 1.60)	0.70		
**Attended training on malaria**					
No	39	1			
Yes	26	1.04 (0.68, 1.60)	0.84		

## Discussion

The present results show that appropriate treatment for malaria provided in drug shops is low; with a third of patients receiving appropriate treatment, as defined by microscopy examination of a blood sample taken at exit. At the same time, the proportion of non-parasitaemic patients who receive unnecessary anti-malarial drugs is high. The factors attributed to this scenario are that diagnosis of malaria in drug shops was primarily presumptive (without laboratory confirmation); few drug shop staff had received training on management of malaria; few knew that Coartem^®^ was the first-line anti-malarial drug; few shops had adequate guidelines on management of malaria; few knew what an RDT was used for; and there was little supervision. The consequences of inappropriate treatment were evident in the fact that many patients in the exit survey had previously sought treatment at another drug shop. In addition to the public health concern of increasing severity of symptoms due to ineffective or inadequate treatment, the economic burden to households of repeated treatment seeking cannot be overlooked. The findings in the present study emphasize the need for implementing interventions aimed at improving appropriate treatment of malaria in drug shops.

The present findings are consistent with another recent study in Uganda that has shown that few (10.3%) febrile children treated at drug shops received appropriate treatment for malaria, and also that few children with both cough and fast breathing received amoxicillin. Similarly, few children with diarrhea received oral rehydration salts, and none received zinc tablets. The study concluded that management of common childhood illness at private sector drug shops in rural Uganda was largely inappropriate [30].

A recent study in Cameroon found that 45% of patients at medicine retailers received ACT, 29% were confirmed with malaria while 70% negative patients received an anti-malarial drug and also concluded that there was inappropriate treatment of malaria at these outlets [31]. Another study in Nigeria reported that ACT were available in 44.7% of private facilities, but that few used ACT for the treatment of malaria, more frequently using SP, chloroquine and artemisinin monotherapy drugs [32].

The findings of the present study suggest that private providers, especially the lower cadre staff, may be overlooked in training them on treatment guidelines and/or other professional development. In many countries, private providers are an integral component of the malaria control strategy to increase access to prompt effective treatment [33]. However, for this strategy to be fully effective the professional knowledge of private providers should be up-to-date and their practice should be in line with national policy. It has recently been shown that availability of ACT the private sector in Uganda is now over 65% [34], but the findings illustrate how the goal of access to prompt and effective treatment cannot be achieved without training drug shops on the use RDTs to guide treatment and updating their knowledge on appropriate management of malaria. These two measures could tremendously improve management of malaria, especially in rural areas where microscopy is non-existent.

This study documents the wide use of anti-malarial drugs among patients with fever at drug shops (Table 4) especially quinine yet this is not a first-line anti-malarial drug. The continued use of non-ACT drugs is a concern for the patient who may be receiving an ineffective treatment, but may also have implications on drug resistance, especially for SP, the recommended drug for malaria prevention in pregnancy (IPTp). There is pressing need to educate the drug shops about the necessity of appropriate treatment of malaria according to national policy. It is further recommended that the National Dug Authority and the District Drug Inspector should intensify supervision of drug shops on this aspect.

Previous studies have also documented anti-malarial drug over-use at drug shops and recommendations to improve targeting malaria patients and rational drug use have been made [35-37]. The findings that most patients came to the drug shop in person, indicates that diagnostic testing of a patient blood sample should be possible in the majority of cases. Recent studies in Uganda have found that drug shops have a desire to stock RDTs and use them to guide treatment; and that most of them can store, administer and dispose of RDTs properly and charge mark-ups similar to those charged on common medicines [38].

In conclusion, the proportion of malaria patients receiving appropriate treatment at registered drug shops in Uganda is unacceptably low. This is due to a combination of presumptive diagnosis of malaria without laboratory confirmation; inadequate training on malaria management and inadequate knowledge that Coartem^®^ is the first-line anti-malarial drug.

## Competing interests

The authors declare that they have no competing interests.

## Authors’ contributions

AKM, SC, KSH and PM conceived the study. All authors participated in the design and implementation of data collection activities, SL and BC undertook the data analysis. AKM wrote the first draft of the manuscript and all authors contributed to, read and approved the final manuscript.
